# When gaze conflicts with space: Implicit eye contact and the reversed congruency effect

**DOI:** 10.3758/s13423-026-02897-5

**Published:** 2026-03-30

**Authors:** Andrea Marotta, Jeannete Chacón-Candia

**Affiliations:** Department of Experimental Psychology, and Mind, Brain, and Behavior Research Center (CIMCYC), University of Granada, Campus Cartuja, 18071 Granada, Spain

**Keywords:** Eye-gaze, Spatial interference, Reversed congruency effect, Eye-contact hypothesis, Social attention

## Abstract

The human ability to perceive and interpret others’ gaze direction is crucial for social interaction and may rely on specialized attentional mechanisms. A particularly striking finding in this domain is the reversed congruency effect (RCE), in which responses are faster when gaze direction and spatial position are incongruent. Two experiments (44 participants each) tested how gaze deviation modulates this effect to test two competing accounts: the eye-contact hypothesis, which attributes the RCE to implicit misperceptions of direct gaze, and the joint-attention hypothesis, which links it to shared attention toward a common focus. Participants performed a spatial interference task in which faces displayed either partially or fully averted gaze. A robust RCE emerged for partially averted gaze, while the effect was reduced for fully averted ones, a pattern that constrains competing accounts of the RCE. Subjective ratings confirmed that subtle deviations were perceived as more direct, but congruency did not affect explicit judgments of being looked at. This dissociation suggests that the RCE may reflect implicit processes related to the social interpretation of gaze rather than explicit awareness, providing constraints on competing accounts of gaze-based attention.

**Supplementary Information:**

The online version contains supplementary material available at 10.3758/s13423-026-02897-5.

## Introduction

Humans are remarkably sensitive to the gaze of others. The simple tendency to follow another person’s eyes, whether it is a passerby glancing across the street or a model in an advertisement, illustrates how gaze direction functions as a powerful social cue. Rather than being a neutral signal, it conveys rich information about others’ intentions, emotions, and focus of attention, shaping how we understand and interact with others (Baron-Cohen et al., 1997; Emery, 2000). From the first months of life, humans show a strong preference for eyes and faces, and this bias supports the development of core social abilities such as joint attention, theory of mind, language, and cultural learning (Batki et al., 2000; Tomasello, 1995). This early sensitivity to eye contact forms the foundation of human social cognition, supporting the idea that gaze is not merely a visual feature but a deeply interpersonal signal. Beyond its communicative role, gaze direction has a profound impact on attention (Frischen et al., 2007). Averted gaze automatically triggers spatial orienting, both covertly (Driver et al., 1999) and overtly (Kuhn & Benson, 2007), even when it is irrelevant to the task. Importantly, this orienting response is not purely reflexive: its magnitude and time course depend on the social meaning and intentionality inferred from the stimulus (Dalmaso et al., 2020). Conversely, direct gaze exerts a distinctive influence on perception and affect, known as the *eye-contact effect* (Senju & Johnson, 2009). Mutual gaze enhances face processing (Macrae et al., 2002), increases physiological arousal (Conty et al., 2010), and evokes approach-related motivation (Hietanen et al., 2008). Collectively, these findings suggest that gaze direction engages attentional processes that are deeply intertwined with social cognition (Blakemore et al., 2004; Chacón-Candia et al., 2023).

However, an important question remains: Is gaze processed by general attentional systems simply because of its directional value, or does it engage uniquely social mechanisms related to intention attribution and mental-state inference? This theoretical tension between “domain-general” and “domain-specific” models of gaze processing has become a central issue in social cognitive neuroscience. Several experimental paradigms, including the widely used gaze-cueing task, have failed to reveal robust differences between gaze and non-social directional stimuli in terms of behavioral and neural responses (Chacón-Candia et al., 2023). This apparent equivalence challenges the notion that gaze automatically recruits specialized social mechanisms and has motivated the search for more sensitive paradigms capable of isolating the social component of attentional control. In this context, an especially intriguing phenomenon is the reversed congruency effect (RCE), which emerges most reliably with gaze stimuli and is thought to reflect uniquely social aspects of attentional processing. In this paradigm, gaze direction and spatial location can be either congruent or incongruent, and responses are typically faster when gaze and location are incongruent, a reversal of the standard spatial congruency effect (SCE; Marotta et al., 2018; Narganes-Pineda et al., 2022; Román-Caballero et al., 2021). The RCE has been proposed to reflect the social meaning of gaze rather than its directional value. Supporting this view, the effect is modulated by the emotional expression of the face (Jones, 2015; Marotta et al., 2022) and correlates negatively with social anxiety, an association not found for non-social directional stimuli (Ishikawa et al., 2021). Moreover, the RCE is selectively associated with later ERP components (N2, P3) related to social evaluation and decision making (Marotta et al., 2019), and its emergence during adolescence coincides with the maturation of social–cognitive networks (Aranda-Martín et al., 2022). Together, these findings suggest that the RCE is sensitive to the social relevance of gaze, although the mechanisms behind it remain under debate.

Several theoretical accounts have been proposed to explain the RCE. One influential explanation is the eye-contact hypothesis, which posits that participants may implicitly misinterpret inward-directed gaze in incongruent trials as direct eye contact, thereby increasing the social relevance of the stimulus and facilitating responses (Cañadas & Lupiáñez, 2012; Jones, 2015; Marotta et al., 2018, 2019). This interpretation aligns with evidence for a default tendency to interpret ambiguous gaze as direct, an adaptive bias given the importance of detecting eye contact (Mareschal et al., 2013). Consistent with interpretations emphasizing the social relevance of gaze, the RCE has been shown to vary with individual differences in sensitivity to social gaze. Ishikawa et al. (2021) reported a negative correlation between RCE magnitude and social anxiety, an association not observed for non-social directional stimuli. In addition, Marotta et al. (2022) found that emotional expression modulated the RCE only in individuals with low autistic traits, with larger effects when gaze was paired with positive facial expressions, a pattern that has been interpreted in terms of self-referential processing of gaze.

An alternative explanation was later proposed within a joint-attention framework. According to this account, the facilitation observed in incongruent trials does not arise from self-referential gaze processing, but from the establishment of shared attention between observer and stimulus. Under this view, the RCE should be strongest whenever gaze most clearly aligns the observer’s attentional focus with that of the stimulus, and weakest when such alignment is reduced. In typical RCE paradigms, inward-directed gaze in incongruent trials points toward the central fixation location, which the observer is also attending to, thereby facilitating processing through attentional alignment (Edwards et al., 2020). In contrast, in congruent trials gaze points away from the observer’s focus, disrupting attentional alignment and slowing responses (Hemmerich et al., 2022). More recently, these accounts have been situated within a higher-level looking-vector framework (Hemmerich et al., 2022). According to this framework, gaze and non-social directional stimuli share a common spatial interference mechanism, but gaze additionally engages a gaze-specific social component, the looking vector, that can invert the SCE. Importantly, this account remains agnostic with respect to the functional nature of the looking vector. From this perspective, the eye-contact and joint-attention hypotheses can be understood as alternative proposals regarding what the looking vector represents, namely self-referential gaze processing versus shared attentional alignment.

Importantly, recent evidence has challenged strong versions of both accounts. Narganes-Pineda et al. (2022) showed that the RCE disappears when participants discriminate eye color while ignoring gaze direction, despite the continued presence of gaze stimuli that could potentially elicit implicit eye contact. This finding demonstrates that eye contact per se is not sufficient to generate the RCE. More fundamentally, this result raises a theoretical tension for the eye-contact account: if the RCE reflects self-referential processing triggered by eye contact, it remains unclear why this mechanism would be abolished by a task that preserves gaze information but shifts attentional selection toward a non-directional eye feature. Thus, the eye-contact hypothesis may be not only incomplete but also strongly dependent on task context. The present study does not attempt to resolve this tension. One possibility, to be examined in future work, is that tasks requiring feature-based selection on non-directional eye features (such as eye-color discrimination) may interfere with gaze-direction processing by biasing attention toward local perceptual properties of the eye region rather than toward its spatial orientation. Under this view, the RCE would be expected to be reduced or eliminated in tasks that require selection of non-directional eye features, but preserved in tasks that do not interfere with the processing of gaze direction. Edwards et al. (2020) further demonstrated that the RCE critically depends on fixation location, with a reversal from an RCE under central fixation to a standard congruency effect under peripheral fixation. While this pattern is consistent with an explanation based on joint-attention alignment toward the fixation point, shifting fixation away from the face also reduces the plausibility of interpreting gaze as self-directed, making it difficult to uniquely attribute the change in effect to joint-attention processes alone.

The present study does not aim to resolve this broader theoretical debate, but rather to examine whether the RCE is differentially modulated by the degree of gaze deviation in a way that constrains competing accounts. Using a spatial interference task in which faces display two discrete levels of gaze deviation, either partially or fully averted, while fixation is held constant at the center, we derive opposing predictions from the eye-contact and joint-attention accounts. According to the eye-contact hypothesis, the RCE should be strongest for partially averted gaze, as subtle deviations are more likely to be misperceived as self-directed and to engage self-referential processing. In contrast, the joint-attention hypothesis predicts a larger RCE for fully averted gaze, which most clearly establishes shared attention toward the fixation point. Thus, although both accounts predict modulation of the RCE by gaze deviation, they do so in opposite directions. In addition to response time measures, we included a subjective gaze perception task to assess participants’ explicit interpretations of gaze direction. This measure was not intended as a direct or diagnostic test between the eye-contact and joint-attention accounts, but rather as a complementary manipulation check, aimed at verifying whether partial versus full gaze deviations systematically differed in perceived directness and fixation-oriented gaze, independently of their impact on performance.

## Experiment 1

### Method

#### Participants

Sample size was determined based on the reversed congruency effect (RCE; *dz* ≈ 0.50) and the interaction effect (*ηp*^*2*^ =.54) reported by Marotta et al. (2018). A power analysis using G*Power 3.1 (Faul et al., 2009) indicated that 34 participants would be sufficient to detect an effect of *dz* = 0.50 in a paired-samples *t-*test (α =.05, power =.80). Complementary simulations using the *Superpower* R package (Caldwell et al., 2020) confirmed that this sample would yield approximately 90% power to detect the expected interaction in a 2 × 2 within-subjects ANOVA. To account for potential data variability inherent to online testing, the target sample size was increased to 44 participants per experiment. The sample consisted of 44 volunteers (42 female), with a mean age of approximately 22.6 years. Participants were undergraduate students from a large public university in Spain, who received course credit for their participation. All reported normal or corrected-to-normal vision and provided informed consent in accordance with the guidelines of the Declaration of Helsinki.

#### Procedure

**Fig. 1:**
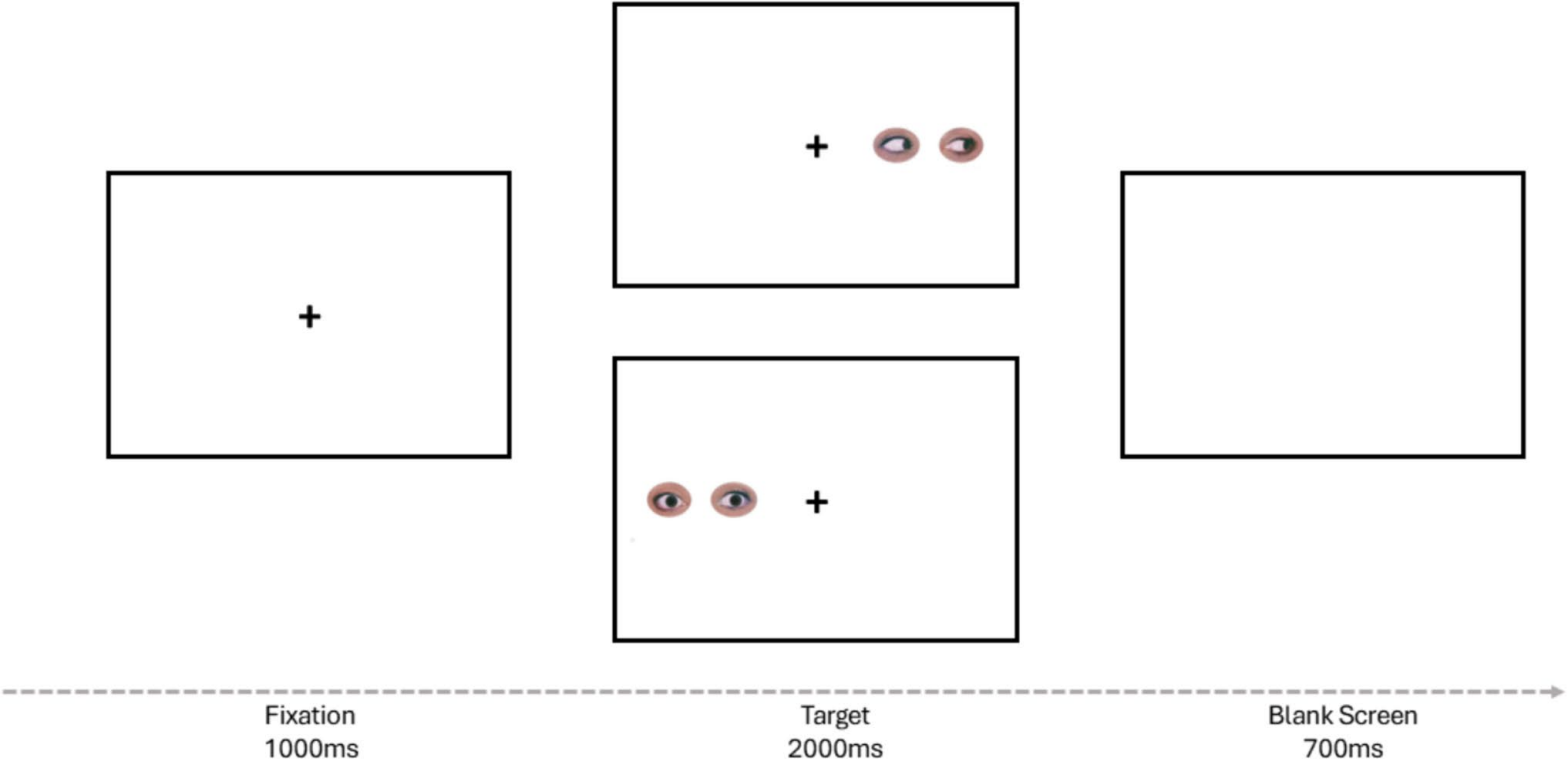
Schematic view of the trial sequence for full and partial gaze deviation target conditions. The example represents: a full-averted gaze target/congruent trial (upper panel), and a partial-averted gaze target/incongruent trial (lower panel)

The experimental task used in this study was developed with the graphical experiment builder OpenSesame (Mathôt et al., 2012). The experiments were conducted and participants were recruited online through the university’s research participation platform. Participants completed the study on their personal computers, and were instructed to ensure a quiet environment during the task. The experiment followed three phases:

1. **Consent and instructions.** Participants provided informed consent, were briefed on the study’s structure, and were asked to complete the task on a desktop or laptop with stable internet.
2. **Spatial interference task.** Participants discriminated, as quickly and accurately as possible, the direction of gaze (left or right) in cropped eyes presented either to the left or right of a central fixation cross. Each trial began with a black fixation cross (30 × 30 px) displayed for 1,000 ms, followed by a pair of eyes (283 × 84 px) shown laterally for up to 2,000 ms or until response (see Fig. 1). Gaze deviation was either partial or full, pointing left or right. In the partial-aversion condition, the iris was displaced by approximately 30–40% of the maximum horizontal range, resulting in a near-direct gaze, whereas in the full-version condition the iris was displaced close to the lateral edge of the sclera (approximately 75–80%). The inner edge of the stimulus was positioned approximately 420 px from fixation. Trials were congruent when gaze direction and presentation side matched (e.g., gaze right, location right) and incongruent when they diverged (e.g., gaze left, location right). Participants pressed “Z” for leftward and “M” for rightward gaze. The 2 (gaze direction: left/right) × 2 (gaze deviation: partial/full) × 2 (presentation side: left/right) factorial design produced 80 trial types distributed across two blocks, for a total of 160 experimental trials. A brief practice block of 15 trials familiarized participants with the task, with visual feedback provided after each trial. Gaze-deviation types (partial vs. full) were intermixed within blocks.
3. **Subjective gaze perception task.** The same stimuli were presented again, comprising four main gaze conditions – partially congruent, partially incongruent, fully congruent, and fully incongruent – each shown on both the left and the right sides of fixation (eight stimuli in total per question). Participants rated each stimulus on two questions: (1) How much do you feel that the eyes are looking at the fixation point? (2) How much do you feel that the eyes are looking at you? Ratings were provided on a 5-point Likert scale (1 = not at all, 5 = completely) and responses were typed directly on the keyboard.

#### Design

The spatial interference task used a 2 × 2 within-subjects design with factors Gaze Deviation (partially vs. fully averted) and Congruency (congruent vs. incongruent). Mean reaction times (RTs) and accuracy were analyzed via repeated-measures ANOVA.

In addition, following reviewer suggestions, complementary distributional analyses were conducted on RTs using vincentized quantiles and linear mixed-effects models to examine whether the modulation of the RCE by gaze deviation depended on response latency. For the subjective ratings, normality was assessed with the Shapiro–Wilk test. Because most distributions violated normality, non-parametric analyses were used: Friedman tests for overall effects and Wilcoxon signed-rank tests for planned comparisons. Planned Wilcoxon comparisons were corrected for multiple comparisons within each question using a Bonferroni procedure (α =.0125). All effects reported as significant survived correction unless otherwise noted.

### Results

#### Spatial interference task

**Fig. 2:**
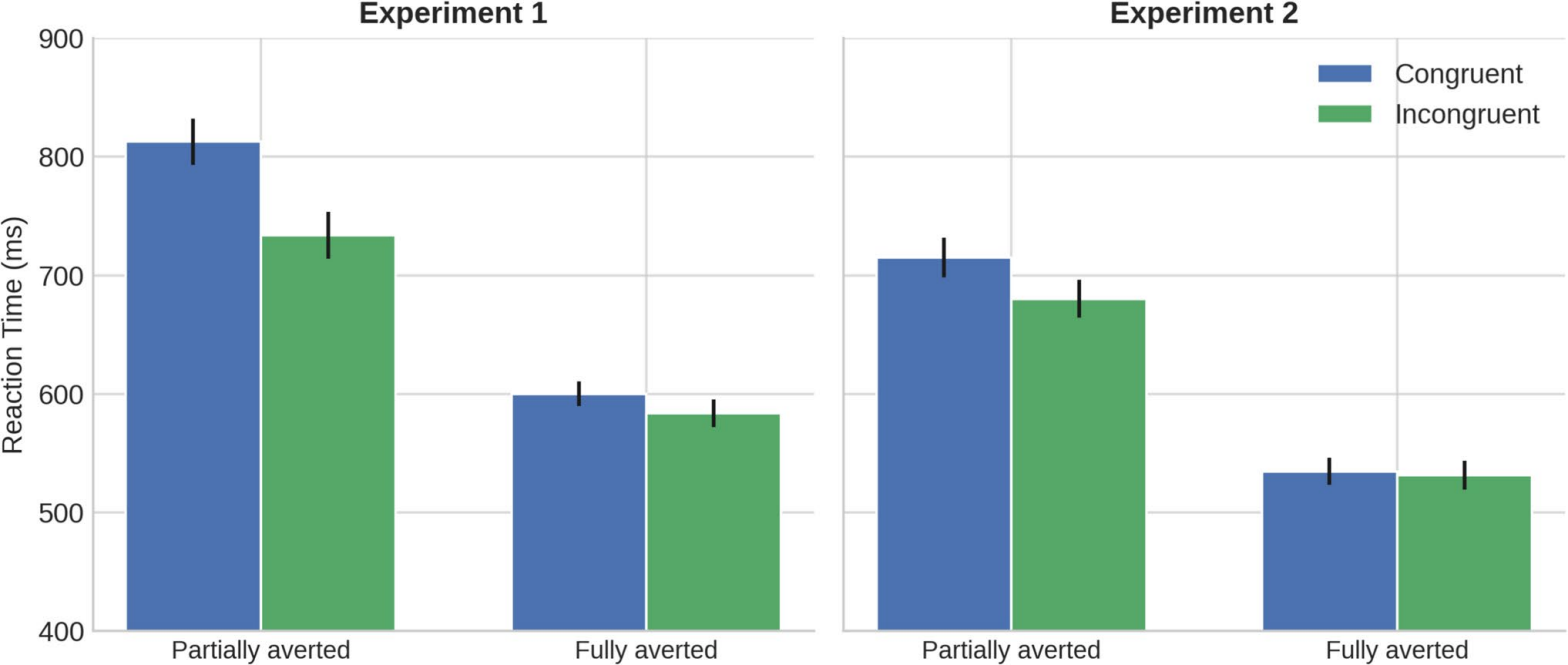
Mean reaction times (± SEM) for congruent and incongruent trials as a function of gaze deviation (partially vs. fully averted) in Experiment 1 and Experiment 2

RTs shorter than 200 ms (0.02%) and longer than 1,300 ms (3.28%) were excluded. This upper cutoff corresponds to standard trimming criteria commonly adopted in a spatial interference task and was used to exclude unusually delayed responses. Mean RTs and accuracy for all conditions are shown in Table 1. ANOVA revealed significant main effects of Gaze Deviation, *F*(1, 43) = 258.91, *p* <.001, *ηp*^*2*^ =.86, and Congruency, *F*(1, 43) = 18.80, *p* <.001, *ηp*^*2*^ =.31, with faster responses for fully averted (*M* = 592 ms) than partially averted gaze (*M* = 773 ms), and for incongruent (*M* = 659 ms) than congruent trials (*M* = 706 ms). Crucially, the interaction was significant, *F*(1, 43) = 18.28, *p* <.001, *ηp*^*2*^ =.30 (see Fig. 2). Planned comparisons showed significant RCEs for both deviations, though stronger for partially averted gaze, *F*(1, 43) = 21.04, *p* <.001, *ηp*^*2*^ =.33, than for fully averted gaze, *F*(1, 43) = 5.13, *p* =.028, *ηp*^*2*^ =.11. Accuracy mirrored these results, with higher accuracy for fully averted (86.5%) than partially averted (85.2%) gaze, *F*(1, 43) = 53.71, *p* <.001, *ηp*^*2*^ =.55, and better performance on incongruent (86.2%) than congruent (85.5%) trials, *F*(1, 43) = 11.62, *p* <.001, *ηp*^*2*^ =.21. The interaction was significant, *F*(1, 43) = 10.80, *p* =.003, *ηp*^*2*^ =.20, with the congruency effect appearing only for partially averted gaze, *F*(1, 43) = 17.18, *p* <.001, not for fully averted gaze, *F* < 1.

**Table 1:** Mean reaction times (RTs, in ms; standard deviations in parentheses) and accuracy (ACC, in %) as a function of gaze deviation and congruency in Experiment 1 and Experiment 2

Condition	Experiment 1		Experiment 2	
	RT	ACC	RT	ACC
Partially averted – Congruent	812.61 (19.44)	84.59	714.94 (16.66)	75.34
Partially averted – Incongruent	733.87 (19.66)	85.86	680.13 (16.01)	89.58
Fully averted – Congruent	600.1 (10.45)	86.49	534.76 (11.26)	95.94
Fully averted – Incongruent	583.74 (11.82)	86.6	531.52 (12.17)	95.48

#### Distributional analysis

To examine whether the modulation of the RCE by gaze deviation depended on response latency, we conducted vincentized distributional analyses using five RT quantiles (10th–90th percentiles) and linear mixed-effects models. For RCE values, the analysis revealed a significant main effect of gaze deviation, *F*(1, 380.76) = 100.84, *p* <.001, indicating a larger RCE for partially averted than for fully averted gaze. Neither the main effect of quantile, *F*(4, 380.11) = 2.03, *p* =.09, nor the deviation × quantile interaction, *F*(4, 380.11) = 0.25, *p* =.91, reached significance, indicating that the enhanced RCE for partially averted gaze was expressed uniformly across the RT distribution. Complementary analyses on raw RTs revealed significant main effects of gaze deviation, *F*(1, 811.05) = 1306.33, *p* <.001, congruency, *F*(1, 811.05) = 86.98, *p* <.001, and quantile, *F*(4, 810.98) = 628.86, *p* <.001. Crucially, the deviation × congruency interaction was significant, *F*(1, 811.05) = 37.18, *p* <.001, confirming that the congruency effect differed as a function of gaze deviation. The deviation × quantile interaction was also significant, *F*(4, 810.98) = 37.21, *p* <.001. In contrast, neither the congruency × quantile interaction, *F*(4, 810.98) = 0.72, *p* =.58, nor the three-way interaction, *F*(4, 810.98) = 0.07, *p* =.99, was significant. These findings indicate that the modulation of the congruency effect by gaze deviation was stable across the RT distribution.

#### Subjective gaze perception task

Means and standard deviations of subjective gaze ratings for each question and experimental condition are reported in Table S1 (Online Supplementary Materials). The Friedman test revealed significant overall differences across conditions for both questions (Fixation: χ^2^(3) = 62.73, *p* <.001; Direct gaze: χ^2^(3) = 74.77, *p* <.001). For the question “How much do you feel the eyes are looking at the fixation point?”, planned Wilcoxon tests showed that ratings were higher for incongruent than congruent trials in both deviation levels (partially averted: *Z* = –4.10, *p* <.001; fully averted: *Z* = –5.17, *p* <.001). Participants also rated partially averted incongruent gaze as less directed toward fixation than fully averted incongruent gaze (*Z* = –4.78, *p* <.001), whereas the difference between the two congruent conditions was nonsignificant (*Z* = –0.21, *p* =.83). For the question “How much do you feel the eyes are looking at you?”, fully averted incongruent gaze was perceived as more direct than fully averted congruent gaze (*Z* = –3.67, *p* <.001), but no difference emerged between partially averted congruent and incongruent conditions (*Z* = –0.47, *p* =.64). Moreover, both partially averted conditions, congruent and incongruent, were judged as significantly more direct than their fully averted counterparts (*Z*s > –4.30, *p*s <.001). All effects reported as significant survived Bonferroni correction.

### Discussion

The behavioral results revealed a clear modulation of the RCE by gaze deviation: the effect was significantly stronger in the *partially averted* condition than in the *fully averted* one. This pattern is compatible with interpretations emphasizing the role of eye contact in enhancing the social salience of the stimulus. Partially averted gaze may be more easily misperceived as direct, increasing their social salience and facilitating faster responses on incongruent trials. When gaze direction deviates only slightly from the observer, it may trigger an implicit sense of being looked at, enhancing attentional engagement despite spatial incongruency. This interpretation should be considered as one possible account among others, rather than as a definitive explanation of the effect. Accuracy mirrored the RT results, with higher accuracy for fully averted than for partially averted gaze and better performance on incongruent than on congruent trials. These convergent findings indicate that the RCE reflects genuine facilitation rather than a simple speed–accuracy trade-off. However, the subjective perception data did not fully align with the behavioral pattern. According to the eye-contact hypothesis, participants should perceive the *partially averted incongruent* condition as more directed toward them than the *partially averted congruent* condition, since incongruent trials involve gaze pointing toward the observer’s side. However, no such difference was observed. Participants rated *partially averted* faces as more direct overall than *fully averted* ones, but congruency had no impact on perceived directness. This dissociation suggests that while behavioral responses are compatible with enhanced self-referential processing of gaze, this effect may operate at an implicit level, without influencing conscious perception. In this sense, the RCE may reflect an implicit process related to the social interpretation of gaze that is not captured by explicit ratings.

## Experiment 2

The main objective of this second experiment was to replicate the findings of the initial study while addressing certain limitations in the subjective gaze perception task. In the first experiment, the spatial interference task revealed a robust reversed RCE, particularly in the partially averted gaze condition, a pattern compatible with interpretations emphasizing eye-contact-related processing. However, the subjective ratings did not fully mirror this pattern, showing no congruency effect in the perceived directness of gaze. This raised the possibility that the explicit rating scale used might not have been sensitive enough to detect subtle differences in gaze perception. Therefore, the second experiment aimed to replicate the RCE pattern observed behaviorally, while refining the subjective perception task to capture more subtle variations in explicit gaze interpretation. To this end, the rating scale was expanded from 5 to 9 points. This modification was motivated by the results of Experiment 1, which revealed reliable effects of gaze deviation on explicit judgments but no modulation by spatial congruency. To rule out the possibility that this null effect reflected limited scale sensitivity rather than a genuine dissociation between implicit and explicit processes, a finer-grained scale was adopted. In addition to refining the subjective measures, Experiment 2 also introduced a procedural change in the spatial interference task. Specifically, gaze deviation was manipulated in separate blocks rather than being intermixed across trials. Blocking gaze deviation increases contextual predictability and reduces the need for trial-by-trial control over directional information. While increased predictability can enhance standard interference effects, it may also attenuate higher-level modulations that depend on flexible or ambiguous interpretation of social cues. Thus, blocking provided a complementary test of whether the modulation of the RCE by gaze deviation would persist under conditions of reduced cognitive control and increased contextual stability.

### Method

#### Participants

To ensure comparable statistical power and facilitate a direct replication of the interaction observed in Experiment 1, we used the same sample size in the current study (*N* = 44; 38 female; mean age 20.6 years).

#### Apparatus, stimuli, procedure, and design

The stimuli and tasks used in this experiment were identical to those in Experiment 1. However, two key procedural modifications were introduced. First, the response scale used in the subjective gaze perception task was expanded from a 5-point to a 9-point Likert scale, allowing for greater sensitivity in participants’ judgments. Second, instead of presenting the different gaze deviation types (partially and fully averted) intermixed within the same block, stimuli were now presented in separate blocks according to gaze deviation. The design of Experiment 2 was identical to that used in Experiment 1.

### Results

#### Spatial interference task

Mean RTs and accuracy for all conditions are shown in Table 1. RTs shorter than 200 ms (0.05%) and longer than 1,300 ms (2.55%) were excluded. RTs showed main effects of Gaze Deviation, *F*(1, 43) = 148.11, *p* <.001, *ηp*^*2*^ =.77, and Congruency, *F*(1, 43) = 11.92, *p* <.002, *ηp*^*2*^ =.22, as well as a significant interaction, *F*(1, 43) = 16.49, *p* <.001, *ηp*^*2*^ =.28. Planned comparisons revealed a significant RCE only for partially averted gaze, *F*(1, 43) = 20.07, *p* <.001, *ηp*^*2*^ =.32, not for fully averted gaze (*F* < 1). Accuracy analyses showed the same pattern, with higher accuracy for fully averted (95.7%) than partially averted gaze (82.5%), *F*(1, 43) = 35.11, *p* <.001, *ηp*^*2*^ =.45, and for incongruent (92.5%) than congruent trials (85.6%), *F*(1, 43) = 12.29, *p* <.001, *ηp*^*2*^ =.22. The interaction was significant, *F*(1, 43) = 15.15, *p* <.001, *ηp*^*2*^ =.26, again driven by the partially averted condition.

#### Distributional analysis

For RCE values, the analysis revealed a significant main effect of gaze deviation, *F*(1, 385.19) = 71.30, *p* <.001, indicating a larger RCE for partially averted than for fully averted gaze. A significant main effect of quantile also emerged, *F*(4, 385.09) = 2.88, *p* =.025, reflecting modest variations in RCE magnitude across the RT distribution. In particular, a polynomial trend analysis revealed a significant quadratic component, *F*(2, 389.13) = 4.99, *p* =.007, indicating that the RCE followed a modest curvilinear pattern across the RT distribution, peaking at intermediate quantiles. Crucially, the deviation × quantile interaction was not significant, *F*(4, 385.09) = 0.92, *p* =.45, indicating that the enhanced RCE for partially averted gaze was expressed consistently across quantiles. The analysis of raw RT distributions revealed significant main effects of gaze deviation, *F*(1, 814.99) = 1409.93, *p* <.001, congruency, *F*(1, 814.99) = 46.67, *p* <.001, and RT quantile, *F*(4, 814.98) = 637.72, *p* <.001. Critically, the deviation × congruency interaction was also significant, *F*(1, 814.99) = 42.13, *p* <.001, indicating that the congruency effect differed as a function of gaze deviation. In addition, a significant deviation × quantile interaction was observed, *F*(4, 814.98) = 27.25, *p* <.001, reflecting overall differences in RT distributions between partially and fully averted gaze across quantiles. In contrast, neither the congruency × quantile interaction, *F*(4, 814.98) = 0.73, *p* =.57, nor the three-way interaction between deviation, congruency, and quantile, *F*(4, 814.98) = 0.30, *p* =.88, reached significance. These findings indicate that the modulation of the congruency effect by gaze deviation was stable across the RT distribution.

#### Subjective gaze perception task

The Friedman tests again revealed significant overall effects for both questions (Fixation: χ^2^(3) = 71.89, *p* <.001; Direct gaze: χ^2^(3) = 105.08, *p* <.001). For perceived fixation gaze, ratings were higher for incongruent than congruent trials in both deviation levels (partially averted: *Z* = –3.85, *p* <.001; fully averted: Z = –5.66, *p* <.001). Participants also judged partially averted incongruent gaze as less directed toward fixation than fully averted incongruent gaze (*Z* = –5.68, *p* <.001), whereas the difference between partially and fully averted congruent conditions did not reach significance (*Z* = –3.52, *p* =.074). For perceived direct gaze, fully averted incongruent gaze were again rated as more direct than fully averted congruent ones (*Z* = –2.12, *p* =.034), although this contrast did not survive Bonferroni correction for multiple comparisons. No difference emerged between partially averted congruent and incongruent conditions (*Z* = –0.62, *p* =.54). Additionally, both partially averted congruent and incongruent trials were perceived as more direct than the corresponding fully averted trials (*Z*s > –3.45, *p*s <.001).

### Discussion

Experiment 2 successfully replicated the critical interaction between gaze deviation and spatial congruency found in Experiment 1. Specifically, the spatial interference task again revealed a strong RCE in the partially averted condition, but no congruency effect in the fully averted condition. While this absence contrasts with some prior studies, it may be due to the blocked design used here. Presenting gaze types in separate blocks likely increased stimulus predictability and reduced the need for inhibitory control (Hübner & Mishra, 2013) factors that should weaken or eliminate RCEs. In the subjective gaze perception task, the expanded 9-point scale did not uncover any new effects. As in Experiment 1, participants did not differentiate between congruent and incongruent trials in the partially averted condition. This pattern is consistent with the possibility that the behavioral modulation of the RCE reflects implicit processes that are not directly captured by explicit gaze ratings.

## General discussion

The present study set out to clarify the mechanisms underlying the reversed congruency effect (RCE) elicited by eye gaze in a spatial interference task, focusing on how the degree of gaze deviation modulates this phenomenon. Across two experiments, we combined behavioral measures with subjective ratings to test two competing accounts: the eye-contact hypothesis, which attributes the RCE to implicit misperceptions of direct gaze, and the joint-attention hypothesis, which links it to shared attentional focus. Across both experiments, a robust interaction between gaze deviation and spatial congruency emerged. When gaze was only partially averted, participants consistently showed faster responses in incongruent than in congruent trials, a clear RCE. In contrast, this effect was reduced when gaze was fully averted. These findings are compatible with interpretations emphasizing eye-contact-related processing, whereby subtle deviations may be more likely to be misperceived as directed toward the observer, enhancing social salience and facilitating responses on incongruent trials. This interpretation aligns with the well-documented direct-gaze bias, the tendency to interpret ambiguous gaze as directed at oneself (Mareschal et al., 2013; Marotta et al., 2018). Crucially, our results are consistent with previous evidence that has been interpreted in terms of eye-contact-related processing. For example, Ishikawa et al. (2021) demonstrated that the magnitude of the RCE, but not the spatial congruency effect elicited by non-social stimuli (e.g., arrows), was negatively correlated with social anxiety levels, suggesting that individuals who are more sensitive to eye contact and tend to avoid it exhibit a diminished RCE. Moreover, findings from Marotta et al. (2022) showed that individuals with low levels of autistic traits exhibited a stronger RCE, particularly when the gaze was paired with happy facial expressions. This pattern is consistent with the self-referential positivity bias, which posits that positive emotional cues such as smiling faces are more likely to be interpreted as directed toward the self. The increased social relevance of such stimuli may enhance the subjective impression of being looked at and consequently amplify the RCE. In contrast, individuals with high autistic traits, who often show reduced sensitivity to social-emotional cues, did not exhibit such modulation, further supporting the idea that the RCE reflects an implicit attribution of eye contact. Interestingly, the RCE in fully averted gaze emerged only in Experiment 1, where deviation types were intermixed, but not in Experiment 2, where they were blocked. This procedural difference may have altered attentional control demands. When gaze types are intermixed, observers must continuously re-evaluate directional meaning, which may amplify the salience of incongruent stimuli. In contrast, blocked presentation increases predictability and reduces the need for top-down control (Hübner & Mishra, 2013), potentially diminishing the RCE. This suggests that contextual factors such as predictability and control demands can constrain the expression of gaze-driven interference effects, even when social cues are present.

Beyond summarizing the main behavioral effects, these findings have important implications for distinguishing between eye-contact and joint-attention accounts. Subjective judgments of gaze direction revealed a complementary picture. Across both experiments, participants rated partially averted gaze as more direct than fully averted gaze, confirming that subtle deviations elicit stronger impressions of eye contact. Crucially, these explicit judgments did not mirror the behavioral congruency effects: in the partially averted condition, where the RCE was strongest, ratings of perceived directness did not differ between congruent and incongruent trials. Although we initially expected congruency-related differences to also emerge in explicit judgments, the absence of such effects suggests that the processes driving the RCE may operate primarily at an implicit level, probably reflecting automatic processes of social attention that are not fully accessible to conscious introspection. This dissociation is consistent with extensive evidence showing that eye-contact effects often influence attention, arousal, and behavior without necessarily giving rise to conscious awareness or explicit report (Senju & Johnson, 2009). Even when the rating scale was expanded in Experiment 2 to capture finer perceptual differences, this pattern persisted, consistent with the interpretation of the RCE as an implicit, socially driven bias.

Despite these strengths, several limitations of the present study should be acknowledged. A limitation of the present study concerns the temporal resolution and diagnostic specificity of the subjective measures. Subjective ratings were collected after completion of the main task and therefore cannot be linked to individual trials or to moment-to-moment fluctuations in the reversed congruency effect. As a result, although these ratings allowed us to assess participants’ explicit interpretations of gaze direction, toward the observer versus toward the fixation point, they do not provide a direct, online measure of shared-attention or self-referential processes during task performance. Future studies should address this limitation by incorporating trial-by-trial or temporally resolved measures, such as online ratings, confidence judgments, or concurrent physiological indices, to more directly adjudicate between shared-attention and eye-contact-based accounts of gaze-driven interference.

More broadly, these findings speak to current theoretical frameworks of gaze-driven spatial interference. In particular, our findings can be situated within the broader looking-vector framework (Hemmerich et al., 2022), according to which gaze and non-social directional stimuli share a common spatial interference mechanism, while gaze additionally engages a gaze-specific social component capable of reversing standard congruency effects. From this perspective, the present results help constrain the functional nature of this social component by showing that it is modulated by the degree of gaze deviation, with larger effects for partially averted gaze than for fully averted gaze.

It is also important to consider alternative explanations based on domain-general attentional and decisional mechanisms. Recent accounts have proposed that the RCE may emerge under conditions of delayed spatial discrimination, when slower, top-down suppression processes dominate performance (Ponce et al., 2025; Tanaka et al., 2024, 2025). Because partially averted gaze produced longer overall RTs than fully averted gaze in the present study, it was important to assess whether the stronger RCE observed in this condition could simply reflect a general slowing of responses.

To address this possibility, we conducted distributional analyses examining the RCE across the entire RT distribution in both experiments. If the effects of gaze deviation were driven primarily by delayed spatial discrimination or late top-down suppression, the modulation of the congruency effect should selectively emerge or increase in the slower portions of the RT distribution. However, this prediction was not supported. Across both experiments, the larger RCE associated with partially averted gaze was expressed consistently across RT quantiles and the critical interaction between gaze deviation and quantile was not significant. Although the overall magnitude of the RCE showed modest variation across the RT distribution, this variation did not depend on gaze deviation. Thus, the stronger RCE observed for partially averted gaze cannot be explained simply by a general slowing of responses. While our results do not exclude a contributory role of domain-general suppression mechanisms, they indicate that such factors alone are unlikely to fully account for the selective modulation of the RCE by gaze deviation observed here. Instead, these mechanisms may interact with social interpretations of gaze, amplifying or constraining their behavioral expression depending on task context. Future studies could further disentangle these contributions by independently manipulating perceptual ambiguity, attentional load, and social relevance. Overall, our results provide converging evidence that gaze stimuli, particularly when subtly averted, produce systematic modulation of spatial interference effects that are consistent with socially relevant interpretation of gaze. However, the present findings do not conclusively adjudicate between eye-contact, joint-attention, and domain-general accounts. Rather, they suggest that the RCE cannot be explained solely in terms of shared spatial alignment or global response slowing, and that social interpretations of gaze may contribute to its modulation under certain task conditions.

By combining behavioral and subjective measures across two experiments, this study contributes to clarifying how gaze direction modulates attention and delineates the conditions under which social biases emerge in spatial interference tasks. More broadly, the findings suggest that integrating perceptual and social perspectives may be essential for understanding the mechanisms of attentional control, bridging low-level sensory processing with higher-level social cognition.

Taken together, the present findings indicate that gaze-driven spatial interference effects are sensitive to the social relevance of gaze and to contextual factors such as perceptual deviation, predictability, and task demands. While these results do not adjudicate conclusively between eye-contact, joint-attention, and domain-general accounts, they place important constraints on explanations that rely exclusively on shared attentional alignment or non-social suppression mechanisms.

## Supplementary Information

Below is the link to the electronic supplementary material.

Supplementary file1 (DOCX 106 KB)

## Data Availability

The raw and filtered trial-level datasets, experimental materials, and documentation for both studies are publicly available on the Open Science Framework repository at: https://osf.io/s25fb/
