# Supplementary material for: When gaze conflicts with space: Implicit eye contact and the reversed congruency effect
Source: Psychon Bull Rev. 2026 Mar 30;33(4):122. doi: 10.3758/s13423-026-02897-5 (PMC13035646; doi:10.3758/s13423-026-02897-5)
Supplement: Supplementary file 1 — Supplementary file1 (DOCX 106 KB) [file 13423_2026_2897_MOESM1_ESM.docx]

**Table S1**

Mean ratings (standard deviations) of subjective gaze ratings for each question and experimental condition in Experiments 1 and 2

| Condition | Experiment 1 | Experiment 2 |
| --- | --- | --- |
| Fixation point – Partially averted – Congruent | 1.74 (0.94) | 2.84 (1.91) |
| Fixation point – Partially averted – Incongruent | 2.59 (1.01) | 3.69 (1.69) |
| Fixation point – Fully averted – Congruent | 1.68 (1.37) | 2.41 (2.37) |
| Fixation point – Fully averted – Incongruent | 4.50 (1.12) | 8.34 (1.38) |
| Direct gaze – Partially averted – Congruent | 3.32 (1.13) | 6.26 (1.90) |
| Direct gaze – Partially averted – Incongruent | 3.43 (1.04) | 6.01 (1.71) |
| Direct gaze – Fully averted – Congruent | 1.18 (0.55) | 1.41 (0.75) |
| Direct gaze – Fully averted – Incongruent | 1.90 (1.34) | 1.88 (1.51) |

*Note. Values are means with standard deviations in parentheses. Partially averted and fully averted refer to the degree of gaze deviation. Congruent and incongruent indicate the spatial relationship between gaze direction and target location.*
